# 5-Fluorouracil Induces Enteric Neuron Death and Glial Activation During Intestinal Mucositis via a S100B-RAGE-NFκB-Dependent Pathway

**DOI:** 10.1038/s41598-018-36878-z

**Published:** 2019-01-24

**Authors:** Deiziane V. S. Costa, Ana C. Bon-Frauches, Angeline M. H. P. Silva, Roberto C. P. Lima-Júnior, Conceição S. Martins, Renata F. C. Leitão, Gutierrez B. Freitas, Patricia Castelucci, David T. Bolick, Richard L. Guerrant, Cirle A. Warren, Vivaldo Moura-Neto, Gerly A. C. Brito

**Affiliations:** 1 Department of Morphology, Faculty of Medicine, Federal University of Ceará, Fortaleza, Ceará Brazil; 2 Department of Physiology and Pharmacology, Faculty of Medicine, Federal University of Ceará, Fortaleza, Ceará Brazil; 3 Paulo Niemeyer Brain Institute, Federal University of Rio de Janeiro, UFRJ, Rio de Janeiro, Brazil; 4 Department of Anatomy, Institute of Biomedical Sciences, University of São Paulo, São Paulo, SP Brazil; 5 Division of Infectious Diseases and International Health, University of Virginia, Charlottesville, VA USA

## Abstract

5-Fluorouracil (5-FU) is an anticancer agent whose main side effects include intestinal mucositis associated with intestinal motility alterations maybe due to an effect on the enteric nervous system (ENS), but the underlying mechanism remains unclear. In this report, we used an animal model to investigate the participation of the S100B/RAGE/NFκB pathway in intestinal mucositis and enteric neurotoxicity caused by 5-FU (450 mg/kg, IP, single dose). 5-FU induced intestinal damage observed by shortened villi, loss of crypt architecture and intense inflammatory cell infiltrate as well as increased GFAP and S100B co-expression and decreased HuC/D protein expression in the small intestine. Furthermore, 5-FU increased RAGE and NFκB NLS immunostaining in enteric neurons, associated with a significant increase in the nitrite/nitrate, IL-6 and TNF-α levels, iNOS expression and MDA accumulation in the small intestine. We provide evidence that 5-FU induces reactive gliosis and reduction of enteric neurons in a S100B/RAGE/NFκB-dependent manner, since pentamidine, a S100B inhibitor, prevented 5-FU-induced neuronal loss, enteric glia activation, intestinal inflammation, oxidative stress and histological injury.

## Introduction

5-Fluorouracil (5-FU) is an antimetabolite drug used to treat several types of cancer, including breast and colorectal cancer. Mucositis and diarrhea are common side effects of 5-FU-based anticancer regimens^1^, which contribute to the increased costs of hospitalization^2^. Previous studies have reported that several inflammatory mediators are involved in 5-FU-related mucositis pathogenesis, including chemokine (C-X-C motif) ligand 4 (CXCL4)^3^, interleukin-4 (IL-4)^4^, interleukin-1β (IL-1β)^5^, chemokine (C-X-C motif) ligand 9 (CXCL9)^6^, TGF-β^6^, platelet activating factor (PAF)^7^, substance P and serotonin^8^. Persistent GI over-contractility has also been demonstrated to persist, even after inflammation has resolved, suggesting that chemotherapy might affect gut neuronal function^9^.

The enteric nervous system (ENS) is composed of neurons and enteric glial cells (EGCs) that are organized into the following two main networks: the submucosal and myenteric plexuses^10^. Previous studies reported an increase in the expression of glial fibrillary acidic protein (GFAP), a marker of EGCs activation, in rectal-biopsy specimens from patients with ulcerative colitis, Crohn’s disease and infectious colitis (caused by *Clostridioides difficile*)^11^. EGCs have an important function in the GI tract, regulating ion transport^12^ and pathogen recognition since they express toll-like receptors (TLRs)-1-5, -7 and -9^13^. These cells are important sources of chemotactic factors and proinflammatory cytokines, such as TNF-α, interleukin-6 (IL-6)^13^ and S100 calcium-binding protein B(S100B)^14,15^.

S100B is a member of the S100 protein family^16^ and is specifically released by EGCs in the intestine^15–17^. In the central nervous system (CNS), S100B is secreted by astrocytes and it plays a dual role in neurons. At low nanomolar concentrations, it stimulates neuronal survival. However, at high concentrations (micromolar), it causes neuronal cell death^18^. *In vitro* studies have indicated that S100B inhibits intestinal epithelium proliferation^19^. These effects were dependent on binding to receptors for advanced glycation endproducts (RAGE)^18,19^.

RAGE is a cell surface receptor and is a member of the immunoglobulin receptor family^20^. RAGE is expressed by neurons, smooth muscle cells, mesangial cells, EGCs, intestinal epithelial cells, and macrophages^20,21^. Despite the deleterious effects of S100B and RAGE in inflammatory intestinal diseases^22,23^, their roles in antineoplastic drug-induced intestinal mucositis has not been explored.

Here, we investigated whether 5-FU treatment affects the ENS and the participation of the S100B/RAGE/factor nuclear kappa B (NFκB) pathway in 5-FU-induced intestinal mucositis and ENS injury pathogenesis.

## Results

### 5-FU increases S100B protein in GFAP-expressing cells

**Figure 1:**
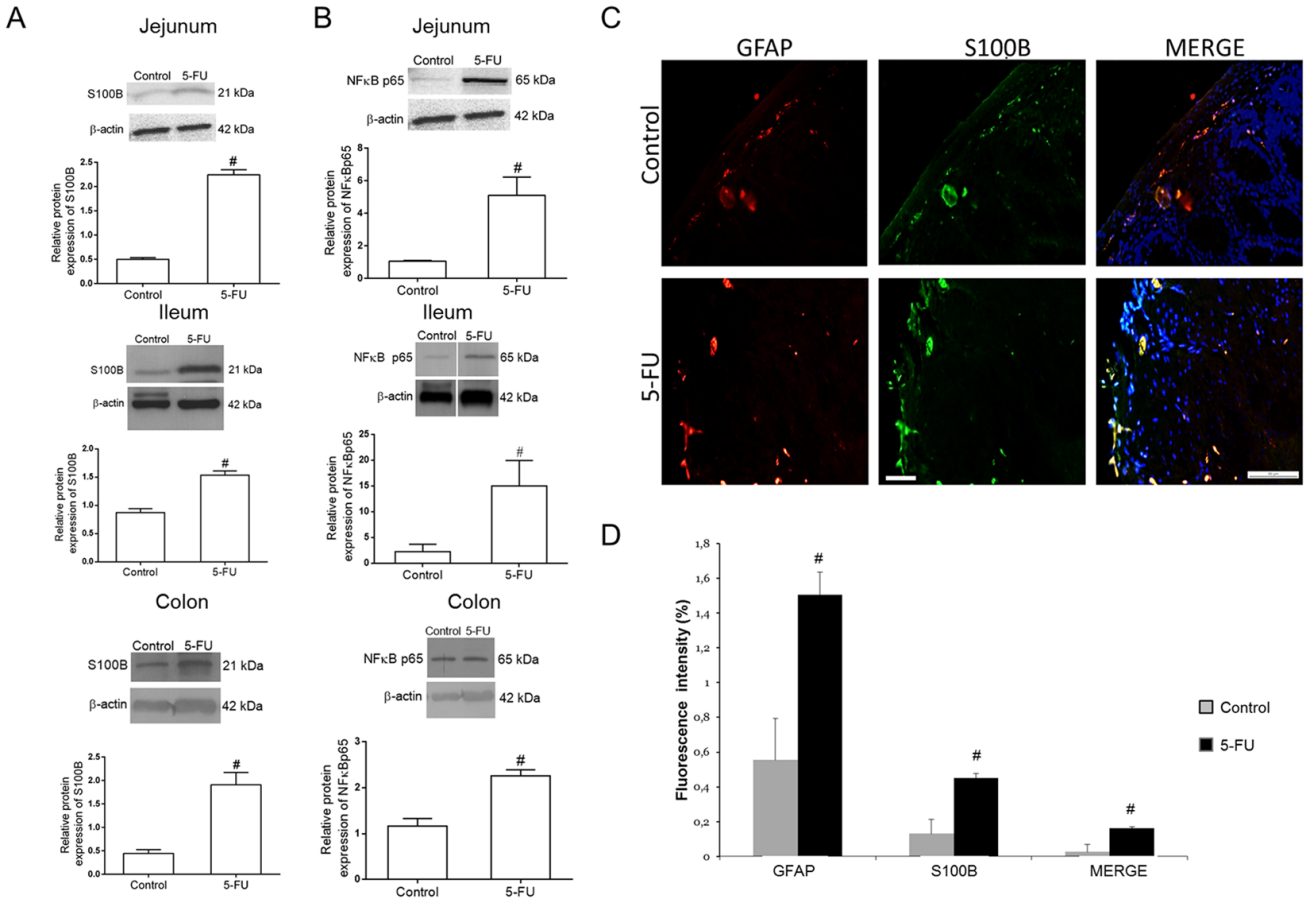
5-FU upregulates S100B and NFκB p65 protein expression and increases GFAP and S100B co-expression in the intestine. (**A**) Representative Western Blot images showing S100B and beta-actin (loading control) protein expression. Quantitative analysis indicates that 5-FU increased the expression of S100B in the jejunum, ileum and colon. Bars represent mean ± SEM for 4 tissue samples in each group. ^#^*P* < 0.01 versus control group, Student’s t test. (**B**) Representative Western Blot images showing NFκB p65 and beta-actin (loading control) protein expression. Quantitative analysis indicates that 5-FU enhanced NFκB p65 protein expression in the jejunum, ileum and colon. Bars represent mean ± SEM for 4 tissue samples in each group. ^#^*P* < 0.05 versus control group, Student’s t test. (**C**) Immunofluorescence images of the colon show GFAP (red) and S100B (green) and their co-localization (Merge, yellow). Nuclei were stained with DAPI. Scale bar, 50 µm. (**D**) Graph represents the mean ± SEM of the immunofluorescence intensity of GFAP, S100B and their co-localization (GFAP and S100B) in colon tissue from 5 fields per mouse from 4 mice in each group. Intensity of fluorescence were quantified using ImageJ. ^#^*P* < 0.05 versus control group, Student’s t test.

We found that 5-FU enhanced (*P* < 0.01) S100B protein expression in the jejunum, ileum and colon compared to the control group (Fig. 1A and S1). S100B protein was expressed in GFAP-positive cells (Fig. 1C). The mean fluorescence intensity of GFAP and S100B was significantly (*P* < 0.05) increased in the 5-FU group compared to the control group (Fig. 1D).

Taken together, these data suggest that 5-FU activates EGCs since GFAP and S100B are glial-cell markers.

### 5-FU increases NFκB p65 protein expression in the intestine

Because S100B is involved in NFκB activation by binding to RAGE, as previously shown in the CNS^18^, we assessed whether 5-FU treatment increases NFκB p65 protein expression in the jejunum, ileum and colon. Consistent with previous studies^24^, we found that 5-FU augmented (P < 0.05) NFκB p65 protein expression in the jejunum, ileum and colon compared to the control group (Fig. 1B and S2).

### The S100B inhibitor Pentamidine reduces 5-FU-related loss of body weight and histopathological injury

Because, S100B is involved in neuron loss and glial activation in CNS through its interaction with RAGE and NFκB activation^18–25^, we hypothesized that S100B is involved in 5-FU-induced glial-cell activation, neuron loss and intestinal mucositis. To confirm this hypothesis, we inhibited S100B by intraperitoneal administration of pentamidine, a S100B inhibitor.

**Figure 2:**
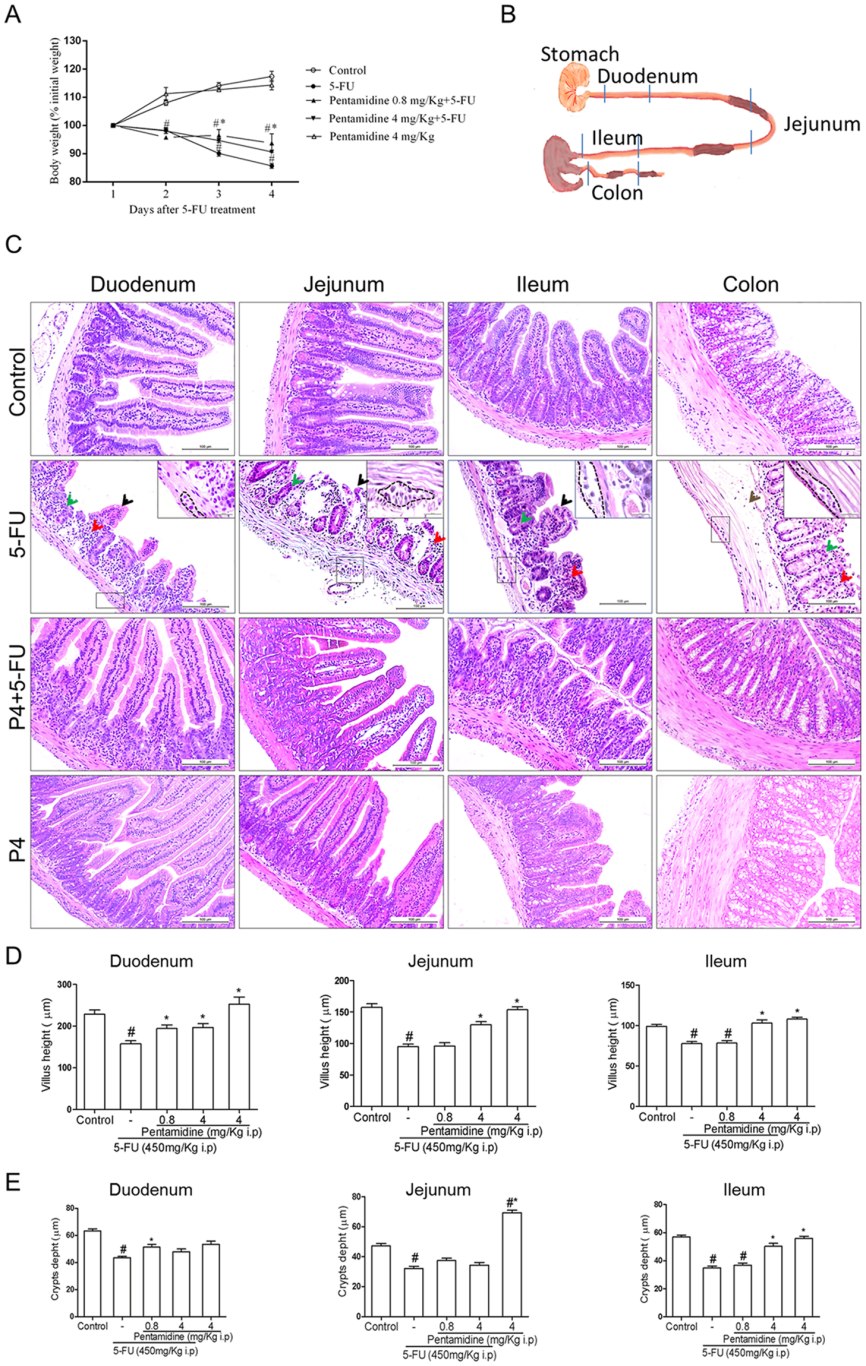
Effects of S100Β inhibition on 5-FU-induced weight loss and histopathological analysis. (**A**) Body weight changes are shown as percentages of the baseline value. Bars represent mean ± SEM of eight mice in each group. ^#^*P* < 0.05 versus control group, *P < 0.05 versus 5-FU group. Two-way ANOVA followed by Bonferroni test. (**B**) Representative scheme of duodenum, jejunum, ileum and colon segments. (**C**) 5-FU induces villi shortening (black arrows), loss of crypt architecture (green arrows) and intense inflammatory cell infiltrate (red arrows) in the duodenum, jejunum, ileum and colon, and edema (brown arrows) in the colon submucosal layer. Dotted line and insert indicate the myenteric plexus. H&E; scale bar corresponds to 100 µm in all figures except for inserts (20 µm). (**D**) Segments of the duodenum, jejunum and ileum were collected for measurement of villus height (10 villi/slide). Bars represent mean ± SEM of 8 mice in each group. ^#^*P* < 0.01 versus control group, *P < 0.01 versus 5-FU group. (**E**) Segments of the duodenum, jejunum and ileum were collected for crypt depth measurements (10 crypts/ slide). Bars represent mean ± SEM of 8 mice in each group. ^#^*P* < 0.01 versus control group, *P < 0.01 versus 5-FU group. One-way ANOVA followed by Bonferroni.

We first investigated whether pentamidine affected 5-FU-induced weight loss and histological alterations. 5-FU caused a pronounced loss of body weight (Fig. 2A), which was partially rescued by a low dose of pentamidine (0.8 mg/kg). Following animal euthanasia, we collected intestinal segments for histopathological analysis (Fig. 2B). We found that 5-FU induced intestinal damage in all intestinal segments investigated, and this damage involved shortened villi, loss of crypt architecture, submucosal edema and a pronounced inflammatory cell infiltrate in the lamina propria (Fig. 2C) compared to the control group. We also observed hypertrophy of myenteric ganglions in 5-FU-injected mice (Fig. 2C). The deleterious effects of 5-FU were also visible in the morphometry of the small intestine, which showed a significant reduction in villus height (Fig. 2D, *P* < 0.01) and crypt depth (Fig. 2E, *P* < 0.01) in all sections evaluated compared to the control group. Pentamidine treatment significantly prevented intestinal injury (Fig. 2C; Fig. 2D, *P* < 0.01; Fig. 2E, *P* < 0.01). Intestinal damage was also measured by blind semi-quantitative analysis (Table 1). All intestinal segments showed moderate to severe intestinal damage in 5-FU-administered mice (Table 1), and pentamidine significantly attenuated this injury in the duodenum and jejunum (*P* < 0.01 vs 5-FU).

**Table 1:** Histological scores for intestinal mucositis.

Intestinal segments	Experimental groups				
	Control	5-FU	5-FU + P0.8	5-FU + P4	P4
Duodenum	0 (0–0)	2 (2–2)^#^	0 (0–1)^#*^	0 (0–1)*	0 (0–0)*
Jejunum	0 (0–0)	3 (3–3)^#^	2 (0–2)^#^	1 (0–2)*	1 (0–1)*
Ileum	0 (0–0)	2.5 (2–3)^#^	1 (1–2)	1.5 (1–2)	0 (0–1)*
Colon	0 (0–0)	3 (3–3)^#^	2 (0–2)	1 (1–2)	0 (0–1)*

Data represent median values (and range) of scores from 0 to 3: Score 0, normal histological findings; Score 1, villus blunting, loss of crypt architecture, sparse inflammatory cell infiltration, vacuolization and edema normal muscle layer; Score 2, villus blunting with fattened and vacuolated cells, crypt necrosis, intense inflammatory cell infiltration, vacuolization and edema and normal muscle layer; Score 3, villus blunting with fattened and vacuolated cells, crypt necrosis, intense inflammatory cell infiltration, vacuolization and edema and muscle layer showing edema, vacuolization and neutrophilic infiltration. Villus alterations were not considered for colon scores. Data were analyzed with Kruskal-Wallis and Dunn’s tests (n = 8). ^#^*P* < 0.01 versus control group, *P < 0.01 versus 5-FU group.

### S100B inhibition reduces 5-FU-induced glial activation, downregulates GFAP and S100B expression in the jejunum and prevents neuron loss

**Figure 3:**
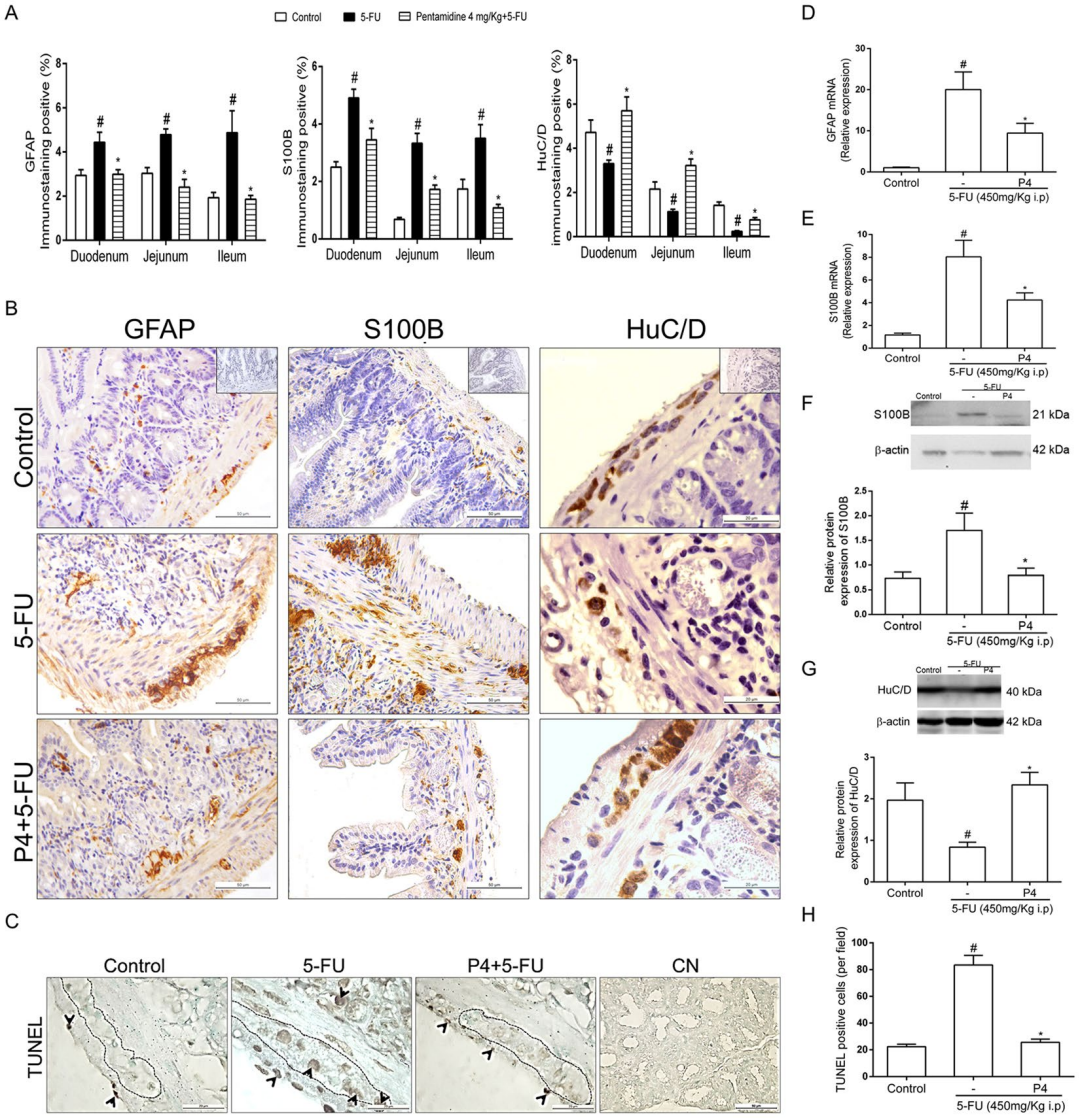
S100B inhibition attenuates 5-FU-induced GFAP and S100B upregulation, reduction of HuC/D expression and cell death in the small intestine. (**A**) Graphs represent the mean ± SEM of the percentage of GFAP, S100B or HuC/D immunopositive area in the small intestine (duodenum, jejunum and ileum) related to total tissue in 5 (GFAP and S100B) or 10 (HuC/D) microscope fields per mouse from 4 mice in each group, quantified using Photoshop. ^#^*P* < 0.01 versus control group, *P < 0.01 versus 5-FU group. (**B**) Representative images illustrating GFAP, S100B and HuC/D immunostaining in the mucosa, submucosa and myenteric plexuses in the jejunum. Scale bar corresponds to 50 µm (GFAP and S100B) or 20 µm (HuC/D). Negative control of each antibody are located on the left of the upper panels (**C**) Representative images illustrating TUNEL-positive cells (black arrows) in the intestinal crypts, smooth muscle layer and myenteric plexus. Scale bar corresponds to 20 µm (except the negative control-CN-50 µm). (**D**) GFAP and (**E**) S100B mRNA expression in the jejunum were evaluated by qPCR (TaqMan® probe). Bars represent mean ± SEM of 6 mice in each group. ^#^*P* < 0.01 (GFAP) or ^#^*P* < 0.01 (S100B) versus control group, *P < 0.01 (GFAP) or ^#^*P* < 0.01 (S100B) versus 5-FU group. (**F**) S100B and (**G**) HuC/D protein expression were evaluated by Western Blot. Bars represent mean ± SEM of 6 mice in each group. ^#^*P* < 0.01 (S100B) or ^#^*P* < 0.01 (HuC/D) versus control group, *P < 0.01(S100B) or ^#^*P* < 0.01 (HuC/D) versus 5-FU group. (**H**) Graphs represent the mean ± SEM of the number of TUNEL-positive cells per field (10 fields per mouse from 4 mice/group) quantified using ImageJ. ^#^*P* < 0.01 versus control group, *P < 0.01 versus 5-FU group. One-way ANOVA followed by Bonferroni.

We next evaluated whether pentamidine decreased 5-FU-induced glial activation in the intestine. We found that 4 mg/kg pentamidine reduced (*P* < 0.01) the percentage of GFAP-immunopositive and S100B-immunopositive area in the small intestine of mice with 5-FU-induced intestinal mucositis compared to the 5-FU group (Fig. 3A). Qualitative analysis showed that 4 mg/kg pentamidine decreased (*P* < 0.01) GFAP and S100B immunostaining in the mucosal, submucosal and myenteric plexuses of the jejunum compared to the 5-FU group (Fig. 3A). To confirm these results, we measured GFAP and S100B mRNA levels by qPCR. GFAP expression was 19-fold higher in 5-FU-treated mice than in controls (*P* < 0.01), whereas pentamidine induced a 10.5-fold decrease (*P* < 0.01) in GFAP mRNA levels in the jejunum compared to the 5-FU group (Fig. 3D). S100B mRNA levels were 6.8-fold higher in 5-FU-treated mice than in the controls (*P* < 0.01), whereas pentamidine induced a 3.8-fold decrease (*P* < 0.01) in S100B mRNA levels in the jejunum compared to the 5-FU group (Fig. 3E). In addition, S100B protein expression was 0.9-fold lower in pentamidine-treated mice with 5-FU-induced intestinal mucositis than in the 5-FU group (*P* < 0.01, Fig. 3F and S3). The treatment with pentamidine alone, caused no changes on S100B expression compared with control group (Fig. S4),

Taken together, these results show that S100B inhibition with pentamidine diminish glial activation in 5-FU-induced intestinal mucositis due to decreased levels of S100B and GFAP in the jejunum.

We also found that 5-FU decreased (*P* < 0.01) the percentage of HuC/D-immunopositive area in the small intestine (Fig. 3A) compared to control animals. Next, we investigated whether S100B inhibition could reduce 5-FU-induced neuronal loss. Immunostaining results showed that 4 mg/kg pentamidine increased (*P* < 0.01) the percentage of HuC/D-immunopositive area in the small intestine of 5-FU-treated mice compared to mice treated only with 5-FU (Fig. 3A). Furthermore, 4 mg/kg pentamidine prevented the loss of neurons in both the submucosal and myenteric plexuses in the jejunum (Fig. 3B, right). To confirm these findings, we performed Western Blot analysis for HuC/D on the jejunum. We found that HuC/D protein expression was 1.13-fold lower in 5-FU-treated mice compared to the control group (*P* < 0.01), whereas HuC/D expression was 1.5-fold higher in pentamidine-treated mice in 5-FU-induced intestinal mucositis than in the 5-FU group (*P* < 0.01, Fig. 3G).

Due to the ability of the S100B inhibitor to prevent 5-FU-induced decreased expression of the HuC/D protein, we evaluated cell death in the intestine by TUNEL assay. We found that 5-FU induced cell death in the intestinal crypts, smooth muscle layer and myenteric plexus (primarily neurons) (Fig. 3C). Quantitative analysis demonstrated that 5-FU enhanced the number of TUNEL-positive cells in the jejunum compared to the control group (*P* < 0.01), whereas 4 mg/kg pentamidine reduced the number of TUNEL-positive cells in 5-FU-induced intestinal mucositis compared to the 5-FU group (*P* < 0.01) (Fig. 3H).

### Higher concentration of S100B but not 5-FU, stimulates enteric neuronal cell death *in vitro*

**Figure 4:**
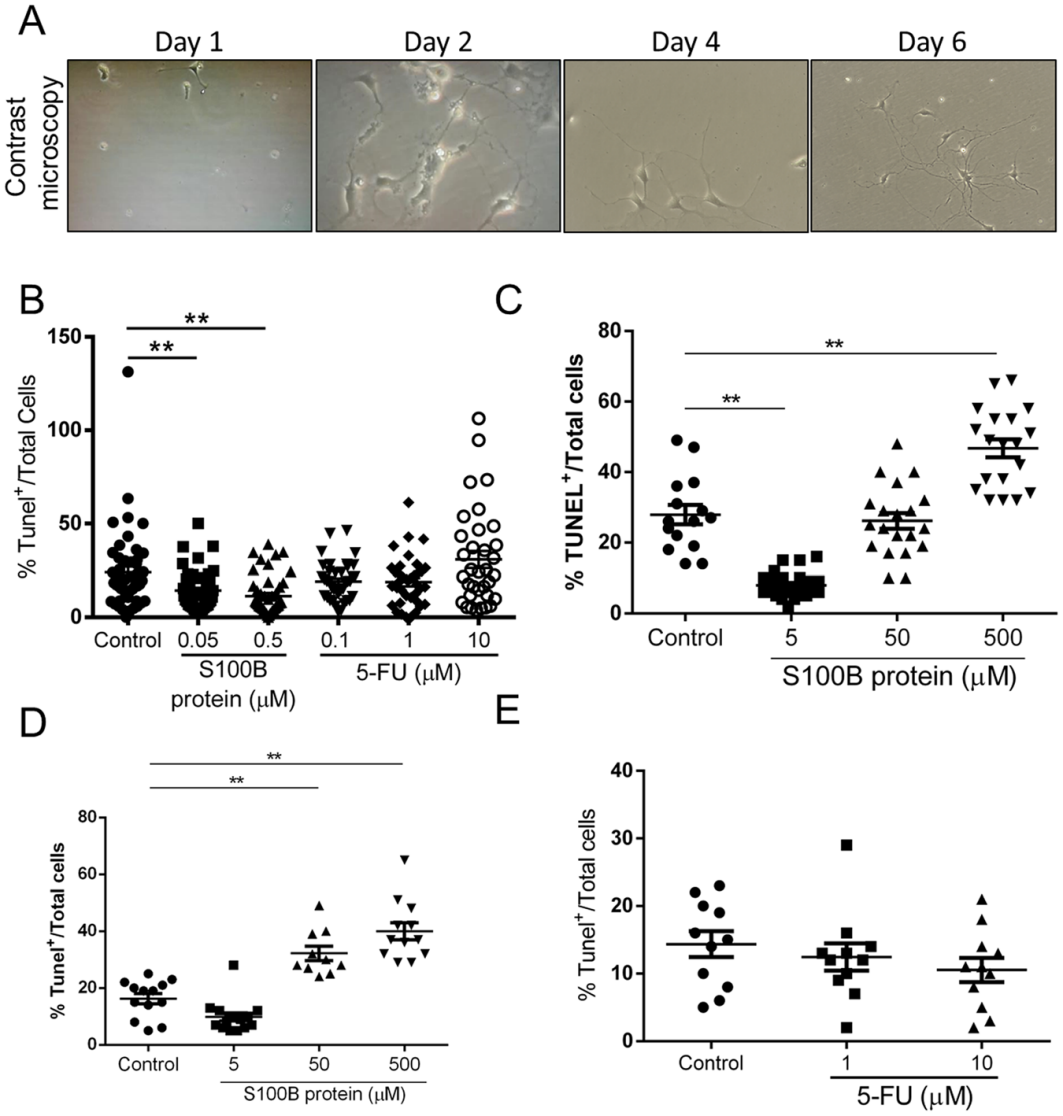
Higher concentration of S100B induces enteric neuronal cell death. (**A**) Representative images of enteric neurons *in vitro* in different time points (day 1–6) in contrast microscope. (**B**) Cells were treated on day 0 with S100B (0.05 µM or 0.5 µM) and 5-FU (0.1 µM, 1 µM or 10 µM) for 24 h. Graph represents the mean ± SEM of the percentage of TUNEL positive cells relative to total cells in eight distinct fields of each well per group from 5 different experiments. (**C**) Cells were treated on day 0 with S100B (5 µM, 50 µM and 500 µM) for 24 h. (**D**) Cells were treated on day 4 with S100B (5 µM, 50 µM and 500 µM) for 24 h. (**E**) Cells were treated on day 4 with 5-FU (1 µM and 10 µM) for 24 h. Graph represents the mean ± SEM of the percentage of TUNEL positive cells relative to total cells in three distinct fields of each well per group from 2 different experiments. All images were analysed using ImageJ software. **P < 0.01. One-way ANOVA followed by Bonferroni.

Because pentamidine decreased the number of TUNEL-positive cells in 5-FU-induced intestinal mucositis, we investigated whether S100B and 5-FU could directly promote enteric neuronal cell death. According to neuronal morphology features showed in Fig. 4A, we incubated enteric neurons with S100B or 5-FU in two different conditions *in vitro* (Days *in vitro* - DIV0 and DIV4). We found that lower concentrations of S100B (0.05 or 0.5 µM) decreased the percentage of TUNEL-positive cells compared to the control group (p < 0.01). However, the treatment with 5-FU did not promote enteric neuronal cell death (Fig. 4B). Given that higher concentrations of S100B have been reported to stimulate neuronal cell death in the CNS, we increased the dosage of S100B *in vitro* to mimic the effect reported in CNS. We found that higher concentration of S100B (500 µM) increased TUNEL-positive cells compared to the controls (p < 0.01) (Fig. 4C).

Similar results were found when investigated the effect of S100B and 5-FU on enteric neurons death (incubating them on day 4) (Fig. 4D,E). However, 50 µM S100B also stimulated (p < 0.01) enteric neurons death compared with control group (Fig. 4D).

### S100B inhibition reduces 5-FU-related RAGE and NFκB expression

**Figure 5:**
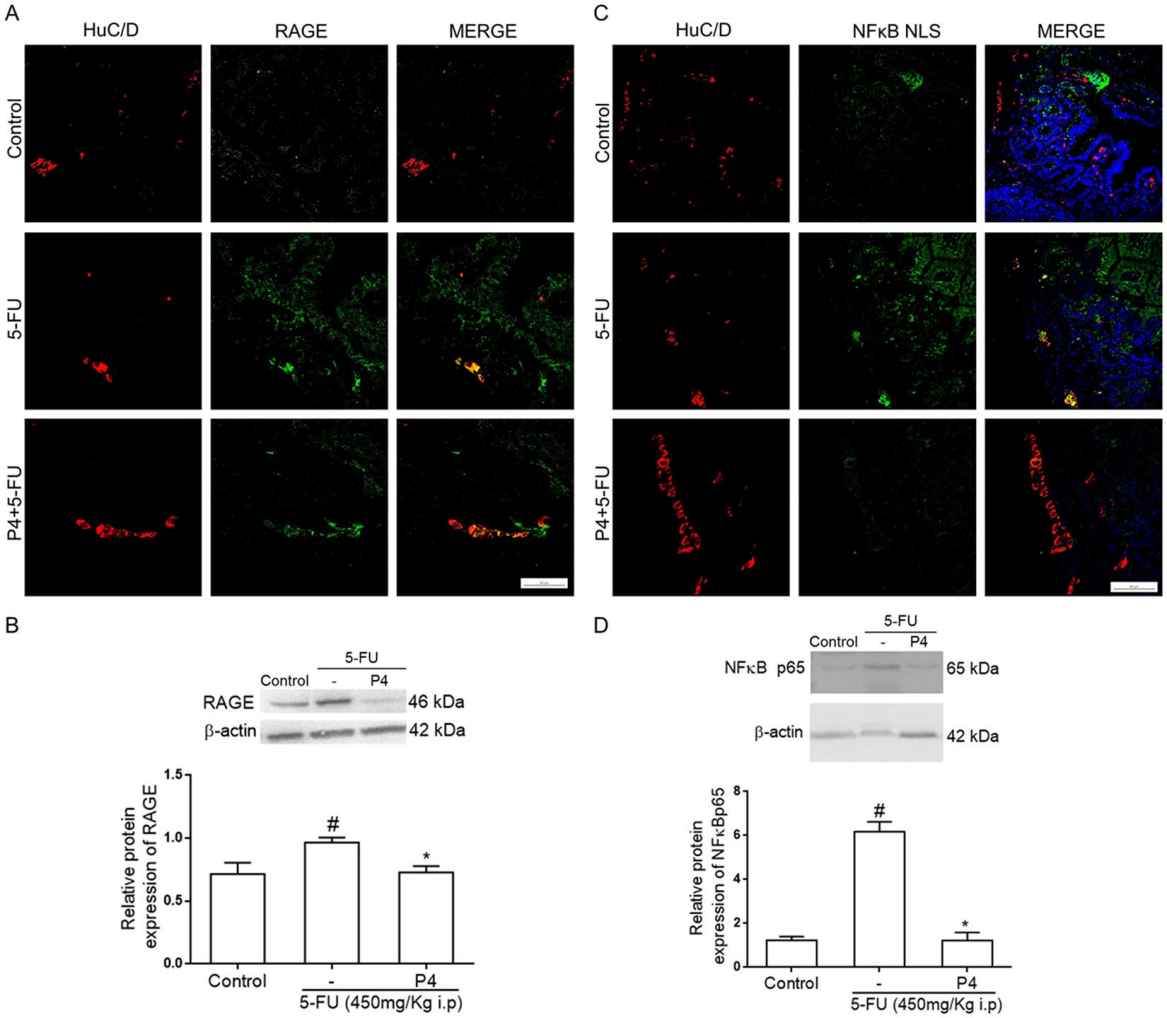
S100B inhibitor decreases 5-FU-induced expression of RAGE and NFκB NLS in enteric neurons and RAGE and NFκB p65 protein expression in the jejunum. (**A**) Jejunal immunofluorescence images demonstrate HuC/D (red) and RAGE (green) expression and their co-localization (Merge, yellow). Nuclei were stained with DAPI. Scale bar, 50 µm. (**B**) RAGE protein expression was evaluated by Western Blot. Bars represent mean ± SEM for 6 tissue samples in each group. ^#^*P* < 0.01 versus control group, *P < 0.01 versus 5-FU group. (**C**) Immunofluorescence images from the jejunum represent HuC/D (red) and NFκB NLS (green) and their co-localization (Merge, yellow). Nuclei were stained with DAPI. Scale bar, 50 µm. (**D**) NFκB p65 protein expression was evaluated by Western Blot. Bars represent mean ± SEM for 6 tissue samples in each group. ^#^*P* < 0.01 versus control group, *P < 0.01 versus 5-FU group. One-way ANOVA followed by Bonferroni.

Because most of the effects of S100B are mediated via RAGE activation, we evaluated the distribution of RAGE within the jejunum and investigated the expression of RAGE by enteric neurons during 5-FU-induced intestinal mucositis. We found that RAGE is expressed at low levels in the jejunum of the control group (Fig. 5A). Interestingly, RAGE is expressed along the myenteric and submucosal plexuses, intestinal epithelium and lamina propria in the jejunum of 5-FU-induced intestinal mucositis mouse model (Fig. 5A). Additionally, RAGE expression overlaps with HuC/D within the myenteric plexus of 5-FU-treated mice. However, 4 mg/kg pentamidine reduced RAGE immunoreactivity within the jejunum and its co-localization with HuC/D in 5-FU-treated mice compared to animals receiving only 5-FU (Fig. 5A).

To confirm these findings, we next examined RAGE protein expression by Western Blot. RAGE protein expression was 0.25-fold higher in the jejunum of 5-FU-treated mice than in controls (*P* < 0.01), whereas its expression was 0.24-fold lower in pentamidine-treated mice with 5-FU-induced intestinal mucositis compared to the 5-FU group (*P* < 0.01, Fig. 5B).

To evaluate whether S100B was required for NFκB activation in enteric neurons during 5-FU-induced intestinal mucositis, we assessed NFκB NLS expression, which is a marker of activated NFκB because the NLS (nuclear localization sequences) appears when NFκB is uncoupled from IκB. NFκB NLS immunostaining was highly increased in 5-FU-treated mice compared to the control group (Fig. 5C). We observed the increase of NFκB NLS immunostaining in enteric neurons of the submucosal and myenteric plexuses, as shown by HuC/D and NFκB NLS co-localization. Furthermore, 5-FU increased NFκB NLS immunostaining in cells from the intestinal epithelium and lamina propria. In contrast, pentamidine decreased NFκB NLS immunostaining in the jejunum of mice with 5-FU-induced intestinal mucositis (Fig. 5C).

We next assessed NFκB p65 protein expression by Western Blot. NFκB p65 protein expression was 4.95-fold higher in 5-FU-treated mice compared to the control group (*P* < 0.01). Inhibition of S100B with 4 mg/kg pentamidine significantly decreased NFκB p65 protein expression in the same proportion compared to mice treated only with 5-FU (*P* < 0.01) (Fig. 5D and S5).

### S100B inhibition decreases the expression of pro-inflammatory cytokines and oxidative stress markers

**Figure 6:**
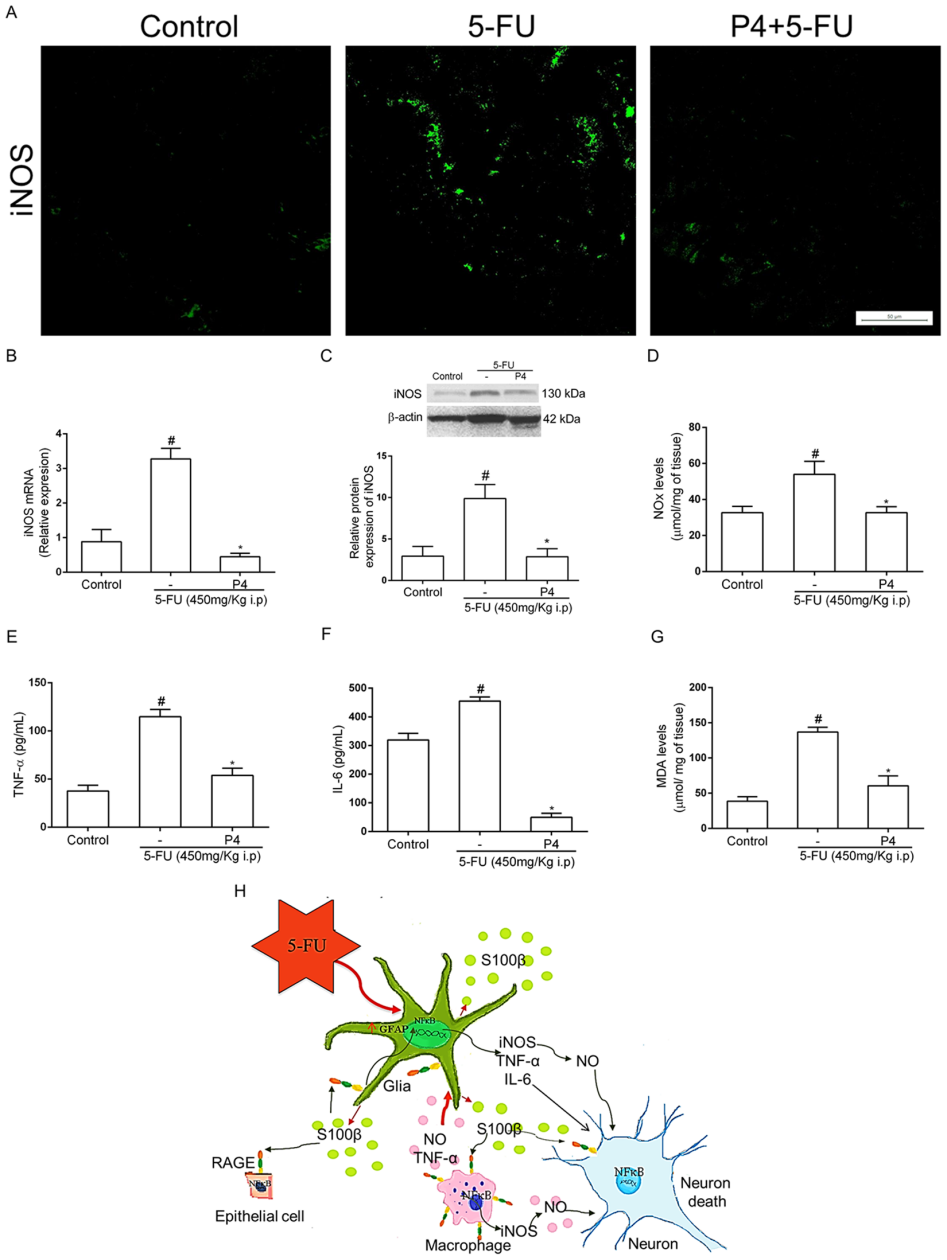
S100B inhibition reduces 5-FU-induced inflammation and oxidative stress. (**A**) Immunofluorescence images from jejunal sections represent iNOS (green). Scale bar, 50 µm. (**B**) iNOS mRNA expression in the jejunum was evaluated by qPCR. Bars represent mean ± SEM of 6 mice in each group. ^#^*P* < 0.01 versus control group, *P < 0.01 versus 5-FU group. (**C**) iNOS protein expression was evaluated by Western Blot. ^#^*P* < 0.01 versus control group, *P < 0.01 versus 5-FU group. (**D**) Nitrite and nitrate levels were evaluated by the Griess method. ^#^*P* < 0.05 versus control group, *P < 0.05 versus 5-FU group. (**E**) TNF-α and (**F**) IL-6 levels were measured by ELISA. (**G**) MDA levels were evaluated by the TBARS method. Bars represent mean ± SEM of 6 mice in each group. ^#^*P* < 0.01 versus control group, *P < 0.01 versus 5-FU group. One-way ANOVA followed by Bonferroni. (**H**) Proposed model of the role of S100B in 5-FU-induced glial cell activation, neuronal loss and intestinal mucositis. 5-FU disrupts the intestinal epithelial barrier leading to EGCs activation by mediators released by macrophages (TNFα, IL-6 and nitric oxide) and bacteria. This process results in increased expression of GFAP and S100B and further release of S100B into the extracellular environment. Additional to that, it also stimulates neuronal death and activates macrophages through the S100B/RAGE/NFκB pathway to release NO, which can also participate in neuronal death.

Inducible nitric-oxide synthase levels were markedly increased in the jejunum of 5-FU-treated mice relative to the control group detected by immunofluorescence (Fig. 6A), qPCR (*P* < 0.01, Fig. 5B), Western Blot (*P* < 0.01, Fig. 6C). Griess reaction also detected an increase in nitrite/nitrate levels on this group (*P* < 0.05, Fig. 6D). We identified an increased iNOS immunostaining in the intestinal epithelium, lamina propria and myenteric plexus (Fig. 6A). Furthermore, pentamidine treatment completely abrogated the expression of this pro-inflammatory enzyme compared to the 5-FU-injected group (Fig. 6A–D).

Moreover, 5-FU also induced a significant increase in the levels of TNF-α (*P* < 0.01, Fig. 6E), IL-6 (*P* < 0.01, Fig. 6F), and MDA (*P* < 0.01, Fig. 6G) in the jejunum. However, pentamidine significantly decreased the level of these proinflammatory markers compared to the 5-FU-treatment (Fig. 6E–G). However, the injection of pentamidine alone did not cause any changes on the levels of nitrite/nitrate, TNF-α and MDA, but decreased the levels of IL-6 (p < 0.01) compared to control group (Fig. S6).

## Discussion

Here, we provide the first evidence of the deleterious effect of S100B on 5-FU-induced intestinal mucositis pathogenesis. We also showed that S100B is produced by EGCs and may be involved in enteric neuron death and reactive gliosis during mucositis as well as in the histological alterations, animal weight loss, inflammatory response and oxidative stress through a RAGE/NFκB-dependent mechanism. Moreover, we showed that high concentrations of S100B, but not 5-FU, causes enteric neuronal cell death *in vitro*, suggesting that S100B mediates the effect of 5-FU on neuronal loss during intestinal mucositis.

5-FU-induced intestinal mucositis animal model is widely used since it reproduces findings in the clinical disease, including intestinal dysmotility, loss of body mass and intestinal inflammation^4–9,26,27^, as observed in colorectal cancer patients undergoing chemotherapy^28^. In this present study, 5-FU caused body weight loss, shortened villi with vacuolated cells and intense inflammatory cell infiltration along the intestinal wall in mice with mucositis relative to the control group. We also demonstrated that 5-FU upregulates S100B protein expression in GFAP-positive cells during mucositis, suggesting that EGCs are an important source of S100B on this model. In the brain, GFAP and S100B upregulation by astrocytes are an indication of reactive gliosis^29^. However, this study is the first to report these findings for 5-FU-induced intestinal mucositis. Previous studies reported that GFAP expression is increased in the inflamed intestinal mucosa of ulcerative colitis patients^11^, although this cell marker is reduced during necrotizing enterocolitis and in the non-inflamed mucosa of Crohn’s disease patients^30^. In the CNS, GFAP is the major intermediate filament protein, and it has been found to control cell shape and movement in mature astrocytes^31^.

Considering the ongoing discussion of the participation of S100B-producing glial cells in different intestinal inflammatory diseases, we investigated the role of S100B signaling in intestinal mucositis induced by 5-FU. Under physiological conditions, the majority of glial cells co-express GFAP, S100B, proteolipidic protein 1 (PLP-1) and SRY-related HMG-box-10 (SOX-10), which are EGCs markers in the adult ENS^15^. Here, S100B was expressed in the mucosal, submucosal and myenteric plexuses of the intestine, as described previously^15–30^. S100B is a calcium-binding protein exclusively secreted by EGCs in the gut wall and it has several intracellular functions including regulating calcium homeostasis^32^, controlling microtubule stability^33^ and modulating mitosis^34^. During inflammation, S100B has been considered a pro-inflammatory cytokine^18^, which signs through RAGE to promote mitogen-activated protein kinase (MAPK) phosphorylation and activation of NFκB, synthesis of pro-inflammatory cytokines and iNOS expression^21–35^. Here, we found that 5-FU increases RAGE and NFκB p65 protein expression within the jejunum, primarily by the intestinal epithelium and enteric neurons, suggesting that S100B may contribute to the intestinal injury during mucositis and to neuronal loss.

To test the role of S100B, we treated mice with the S100B inhibitor pentamidine^36^. Interestingly, pentamidine prevented 5-FU-induced neuronal loss, EGCs activation, intestinal inflammation, oxidative stress and histological injury. The protective inhibitory effect of pentamidine was previously reported in animal models of Alzheimer’s disease, where it prevented neuroinflammation and RAGE activation^37^. In the gut, pentamidine also protected animals against experimental DSS-induced colitis^38^. A previous study showed that inhibition of the metabolic activity of GFAP-positive cells through a gliotoxin (fluorocitrate) reduced motility and transit in the small intestine, indicating that enteric glia modulates enteric neuronal activity^39,40^.

The effect of S100B inhibition on reducing 5-FU-induced weight loss might be associated with increased villus height and consequently increasing the absorptive area at least in the duodenum (0.8 mg/kg). It is known that pentamidine decreases the appetite when administered in major doses, which could explain the reduced effect of 4 mg/Kg pentamidine on weight loss. Patients receiving 5-FU lose body mass due to the reduction of nutrient absorption and diarrhea and changes in intestinal motility. Similar findings have been described in animals^9–27^. We hypothesize that S100B is a key regulatory molecule since its inhibition led to the recovery of intestinal architecture. Previous studies suggest that S100B impairs intestinal epithelial cell proliferation, possibly by binding to and activating RAGE^19^.

Our present findings suggest that 5-FU increases RAGE expression in enteric HuC/D-positive neurons. Several studies have used HuC/D as a pan-neuronal marker of enteric neurons^41–43^. We demonstrated that 5-FU decreased HuC/D expression within the gut wall and induced neuronal death. Neuronal loss may be one factor that contributes to 5-FU-induced altered intestinal contractility, which persists even after active inflammation has been resolved^9^. In accordance with these findings, a pilot study from colonic biopsies of patients after 5-FU treatment showed a decrease in enteric neurons of the myenteric plexus, including morphological and electrophysiological changes in these neurons^44^. McQuade *et al*.^45^ demonstrated that 5-FU decreases the number of nitric oxide synthase-immunoreactive neurons during the inflammatory phase. In ulcerative colitis, Crohn’s disease and necrotizing enterocolitis, enteric neurons cell death has also been observed^30^ and it is likely to contribute to changes in the intestinal motility. We provide evidence that 5-FU-induced neuronal death occurs in a S100B/RAGE/NFκB-dependent manner since pentamidine-treated animals expressed increased HuC/D levels and decreased RAGE and NFκB levels in neurons. In accordance to this hypothesis, we showed that 5-FU was unable to directly cause enteric neuronal death *in vitro*. In contrast, S100B have a dual effect, since higher S100B concentrations caused a significant neuronal death, while lower S100B concentrations showed a protective action.

Notably, pentamidine treatment reduced iNOS expression, IL-6 and TNF-α levels and MDA accumulation in the small intestine. Cirillo *et al*.^21^ showed that the use of antibodies against S100B or RAGE reduces lipopolysaccharide (LPS)- or INF-γ-induced iNOS expression in primary culture of EGCs. Additionally, S100B stimulated iNOS gene expression, production of nitric oxide and TNF-α protein expression in macrophages isolated from the peritoneal cavity in a concentration-dependent manner^46^. Brown *et al*.^41^ suggested that EGC mediate neuronal cell death in colitis through an oxidative stress reaction. Therefore, inhibition of S100B signaling can improve intestinal mucositis, partially by modulating enteric ganglia and reducing pro-inflammatory mediators and oxidative stress.

In conclusion, this study demonstrates an important role for enteric glia in 5-FU-induced intestinal mucositis pathogenesis. EGCs release S100B, which in turn activates NFκB signaling in a RAGE-dependent manner in neurons, leading to neuronal cell death. An autocrine pathway might also induce glial cell expression of pro-inflammatory cytokines, iNOS-derived nitric oxide release and oxidative stress (Fig. 5H). Taken together, these results show that the S100B/RAGE/NFκB pathway may be critical in the pathogenesis of 5-FU-induced intestinal mucositis. Therefore, the S100B signaling pathway appears to be a promising pharmacological target to prevent 5-FU-induced intestinal mucositis and ENS injury.

## Methods

Seventy-six male Swiss mice, weighing 25–30 g (6–8 weeks of age), were housed in temperature-controlled rooms under 12 h light-dark cycles. The animals received water and food ad libitum. Surgical procedures and animal treatments were conducted in accordance with the Guidelines for Institutional and Animal Care and Use of the Federal University of Ceará, Brazil. All procedures involving animals were approved by the Federal University of Ceará Committee on the Ethical Treatment of Research Animals (Protocol No. 80/2015).

### Induction of experimental intestinal mucositis

Intestinal mucositis was induced experimentally as described previously^4,7,26,27^, with some modifications. Briefly, 5-FU (450 mg/kg, IP, single dose) was administered and the mice were euthanized 3 days later.

### Experimental groups

Mice were divided into two experimental groups: Control (healthy mice that received only saline solution, IP) and 5-FU (mice with 5-FU-induced intestinal mucositis). To study the role of S100B in 5-FU-induced ENS alterations and 5-FU-induced mucositis, three groups were added: 5-FU (450 mg/kg, IP) + pentamidine (0.8 or 4 mg/kg, IP) and pentamidine (4 mg/kg, IP). Pentamidine was injected 24 h after the 5-FU dose.

### Western Blot analysis

Intestinal segments (jejunum, ileum or colon) were homogenized in RIPA lysis buffer (25 mmol/L Tris-HCl, pH 7.6; 150 mmol/L NaCl; 5 mmol/L EDTA; 1% NP40; 1% Triton X-100; 1% sodium deoxycholate; 0.1% SDS) and protease inhibitor (1 µL inhibitor: 100 µL RIPA). For protein extraction, intestinal samples were centrifuged (17 min, 4 °C, 13000 rpm) and the supernatant was collected. Protein concentrations were determined through the bicinchoninic acid assay according to the manufacturer’s protocol (Thermo Fisher Scientific). SDS PAGE (10%, 8% or 7%) was performed using 20 µg (S100B, NFκB p65, RAGE, iNOS and β-actin) or 60 µg (HuC/D and β-actin) protein (previously prepared with Laemmli sample buffer - Bio-Rad, and denatured at 95 °C for 5 min or 10 min for HuC/D and its control protein). The protein was transferred to a PVDF membrane (Bio-Rad) for 2 h, blocked with 5% FBS for 1 h, incubated overnight with primary antibodies (rabbit anti-β actin, 04–1116, 1:500, Merck Millipore; goat anti-S100B, sc7851, 1:100; mouse anti HuC/D, A21271, 1 µg/mL, Invitrogen; rabbit anti-RAGE, sc5563, 1:200; rabbit anti-NFκB p65, sc372, 1:200 or rabbit anti-inducible nitric oxide synthase-iNOS-sc8310, 1:100, Santa Cruz Biotechnology) and secondary antibodies (goat anti-rabbit, 656120, Invitrogen, 1:1000; rabbit anti-goat, A16142, Invitrogen, 1:2000; or goat anti-mouse IgG, 626520, Invitrogen, 1:500) for 1 h and 30 min. The membranes were incubated in ECL according to the manufacturer’s instructions (Bio-Rad) and the chemiluminescence signal was detected using the ChemiDoc XRS system (Bio-Rad). Densitometric quantification of bands was performed using ImageJ software (NIH, Bethesda, MD, USA).

### Indirect immunofluorescence

For immunofluorescence assays, intestinal tissue sections were deparaffinized and permeabilized with phosphate buffered saline (PBS)/0.1% Triton X-100. Antigen retrieval was performed by boiling the slides in 0.01 mol/L trisodium citrate buffer, pH 6.0, for 20 min. Sections were pre-incubated in 5% FBS (Sigma-Aldrich) containing 0.2% Triton X-100 and 2% horse serum (Sigma-Aldrich) at room temperature for 30 min. Slides were then incubated overnight at 4 °C with primary antibodies (goat anti-GFAP, Santa Cruz Biotechnology, sc6170, 1:100; rabbit anti-S100B; mouse anti-HuC/D, 1:400; rabbit anti-RAGE, 1:200; rabbit anti-NFκB NLS, Santa Cruz Biotechnology, sc114, 1:200; or rabbit anti-iNOS, 1:100). Sections incubated with antibody diluent without a primary antibody were used as negative controls. After washing in PBS/0.1% Tween 20, slides were incubated with the appropriate secondary antibodies (Alexa fluor 488 donkey anti-rabbit IgG, Invitrogen, A21206, 1:500; Alexa fluor 594 donkey anti-mouse, Invitrogen, A21203, 1:400; or Alexa fluor 647 donkey anti-goat, Invitrogen, A21447, 1:400). Nuclei were then stained with DAPI (4’,6-diamidino-2-phenylindole), and the slides were mounted using Fluoromount (DAKO). Images were acquired with a confocal microscope (LM 710, Zeiss, Germany) using a 40x/NA 1.4 objective. For colon sections, immunofluorescence (IF) intensity was quantified in ImageJ software (NIH, Bethesda, MD, USA).

### Histopathological analysis

Intestine samples were fixed in 10% neutral buffered formalin, dehydrated and embedded in paraffin. Sections (5 µm thick) were obtained for hematoxylin and eosin staining (H&E) and for subsequent light microscopy examination (200x). Intestinal villi and crypt lengths (8 to 10 villi per slide; 6 mice per group) were measured using ImageJ 1.4 (NIH, Bethesda, MD, USA). Mucosal injury was assessed using a modified histopathological score system described previously^47,48^.

### Immunohistochemistry

Sections (4 µm thick) were prepared from paraffin-embedded intestinal tissues. After deparaffinization, antigens were recovered by incubating the slides in citrate buffer (pH 6.0) for 20 min at 95 °C. Endogenous peroxidase was blocked with 3% H_2_O_2_ for 10 min to reduce nonspecific binding. Sections were then incubated with GFAP (IS524, DAKO), S100B (IR504, DAKO) or HuC/D (A21271, Invitrogen, 1 µg/mL) antibodies for 1 h. Sections were then incubated for 30 min with polymer (K4061, DAKO). Antibody binding sites were visualized by incubating the samples with diaminobenzidine–H_2_O_2_ (DAB, DAKO) solution. Sections incubated with antibody diluent without a primary antibody were used as negative controls. Antibody specificity was evaluated using positive controls for GFAP, S100B and HuC/D in the mouse cerebral cortex or for RAGE in the rat liver (data not shown). The amounts of DAB products from immunostaining were estimated from digital images of at least ten different areas of each section (from 4 specimens per group) at 400x (GFAP and S100B) and 1000 × (HuC/D) magnification using Adobe Photoshop software. The percentage of immunopositive area was calculated by dividing the DAB-positive staining (immunostaining-positive pixels) by the number of pixels per total tissue image multiplied by 100, as described previously^49^.

### Terminal Deoxynucleotidyl Transferase-Mediated Deoxyuridine Triphosphate Nick-End Labeling (TUNEL) Assay

Cell death was investigated using a TdT-mediated dUTP nick end-labeling (TUNEL) assay according to the manufacturer’s protocol (ApopTag, S7101, Merck, Millipore). Four micron-thick sections were prepared from paraffin-embedded jejunum segments. After deparaffinization and rehydration, antigens were recovered with 20 μg/mL proteinase K for 15 min at room temperature. Endogenous peroxidase was blocked with 3% H_2_O_2_ for 10 min to reduce nonspecific binding. After washing, sections were incubated in a humidified chamber at 37 °C for 1 h with TdT buffer containing TdT enzyme and reaction buffer. Specimens were incubated for 10 min at room temperature with a stop/wash buffer and then incubated in a humidified chamber for 30 min with anti-digoxigenin peroxidase conjugate at room temperature. After a series of PBS washes, slides were covered with peroxidase substrate to develop color and then washed in three changes of dH_2_O and counterstained in 0.5% methyl green for 10 min at room temperature. TUNEL-positive cells were counted from digital images of at least ten different areas of each section (from 4 specimens per group) at 1000x using ImageJ software (NIH, Bethesda, MD, USA).

### RNA extraction and reverse transcription

Total RNA was isolated using an RNA isolation protocol (PROMEGA). RNA was quantified by NanoDrop (Thermo Fisher Scientific), and RNA quality was determined by examining the 260/280 ratio >1.8. A total of 1 µg RNA was then reverse transcribed using a High-capacity cDNA reverse transcription Kit (Applied Biosystems) according to the manufacturer’s protocol.

### TaqMan qPCR

mRNA expression was analyzed by TaqMan quantitative PCR (qPCR) according to the manufacturer’s instructions using pre-made probes (Applied Biosystems, USA). Sequences or pre-made probe ID numbers are listed in Table 2. To compare gene expression between conditions, the expression (normalized by GAPDH endogenous control) was quantified relative to the control condition. A total of 40 ng cDNA (diluted 5x) was added to the 20x TaqMan gene expression assay (1 µL of S100B, GFAP, iNOS or Gapdh), 2x TaqMan gene expression master mix (10 µL) and RNA-free water (5 µL) to a final volume of 20 µL. Then, 20 µL of PCR reaction mix was transferred to each well. The plate was loaded into the instrument, sealed and centrifuged. The thermocycler parameters were 50 °C for 2 min and 95 °C for 10 min followed by 40 cycles of 95 °C for 15 s and 60 °C for 60 s. All fold changes were calculated by the ΔΔC_t_ method.

**Table 2:** Description of TaqMan® probes used to detect target genes in the experiment.

Genes	
**GFAP (Glial fibrillary acidic protein)**	
ID assay	Mm01253033_m1
TaqMan probe	AGAAAACCGCATCACCATTCCTGTA
Amplicon length:	75
**S100B (S100 protein, beta polypeptide, neural)**	
ID assay	Mm00485897_m1
TaqMan probe	CTTCCTGGAGGAAATCAAGGAGGAGCAG
Amplicon length:	69
**iNOS (Nitric oxide synthase 2, inducible)**	
ID assay	Mm00440502_m1
TaqMan probe	GCCTTGTGTCAGCCCTCAGAGTACA
Amplicon length:	66
**Gapdh (Gliceraldehyde-3-phosphate dehydrogenase)**	
ID assay	Mm99999915_g1
TaqMan probe	GGTGTGAACGGATTTGGCCGTATTG
Amplicon length:	109

### Jejunal nitrite level detection

Jejunal segments were removed for nitrite/nitrate (NO_X_) quantification using the Griess method^50^. First, nitrate was reduced to nitrite by incubation with nitrate reductase and nicotinamide adenine dinucleotide overnight at room temperature. Griess reagent (1% sulfanilamide and 0.1% naphthyl ethylenediamine dihydrochloride in 5% phosphoric acid) was then added and the total nitrite concentration was determined by its 540 nm absorbance value, assigned to the Griess reaction. A calibration curve was obtained by incubating sodium nitrite (10 to 200 µmol/L) with Griess reagent. Nitrate/nitrite levels were expressed as micromoles per milligram of intestinal tissue.

### Jejunal TNF-α and IL-6 levels

Jejunal samples were collected and homogenized in 1% PBS to evaluate TNF-α and IL-6 levels by using a commercial ELISA kit (R&D Systems) according to the manufacturer’s instructions. TNF-α and IL-6 levels in the jejunum were measured as picograms per milliliter of tissue.

### MDA

Lipid peroxidation was measured as malondialdehyde (MDA) production through the thiobarbituric acid-reactive substances (TBARS)^51^ in jejunal segments of mice. Briefly, 250 µL of 10% homogenate of intestinal tissue was mixed with 1.5 mL of 1% H_3_PO_4_ and 0.5 mL 0.6% thiobarbituric acid aqueous solution, and the mixture was stirred and heated in boiling water for 45 min. After cooling, 2 mL of n-butanol was added, and the mixture was homogenized. The butanol layer was separated, and the difference between the optical densities at 535 and 520 nm was used to calculate MDA concentrations, which were expressed as micromoles of MDA per milligram intestinal tissue.

### Culture of enteric neurons

Small intestine LMMPs (longitudinal muscle myenteric plexus) from adult mice were dissected in ice-cold DMEM/F12 12.5 HEPES, 2%FBS, P/S and cut in 1 cm pieces. Samples were incubated in 0.5 mg/ml collagenase IV (Sigma-Aldrich) and 0.5 mg/ml DNase (Sigma-Aldrich) at 37 °C on shaker (60 rpm) for 15 min and centrifuged (200 × g, 5 min at 4 °C). Tissues were washed gently (15 rpm) for 10 min at room temperature and incubated in 1 mL TrypLE in 37 °C shaker (40 rpm) for 10 min and then spun down at 200 × g for 5 min at 4 °C. Remaining tissues were mechanically dissociated using a P1000 pipette. Cells were plated in NeuroBasal Media (GIBCO) containing 2% B27, 1% N2, 1% Glutamax and 1% penicillin/streptomycin onto Laminin/Fibronectin coated dishes. In the following day or three days after culture, cells were treated with 0.05 µM, 0.5 µM, 5 µM, 50 µM ou 500 µM S100B (Sigma S6677) and 0.1 µM, 1 µM and 10 µM 5-FU (Libbs) for 24 h. Samples were fixed with 4% PFA for 10 min at room temperature.

### TUNEL assay for *in-vitro* experiments

Cell death was analyzed by TUNEL assay following the protocol from the manufacturer (ApopTag, S7101, Merck-Millipore or *In situ* cell death detection kit, Fluoroscein, ROCHE). Apoptotic cells were counted from Confocal (Leica SP5) of at least three-eight distinct fields of each well per group from 2–5 different experiments. Cells were counted using ImageJ software (NIH, Bethesda, MD, USA).

### Statistical analysis

Data are presented as the mean ± standard error of the mean (SEM) or as medians when appropriate. Student’s t test, one or two-way Analysis of Variance (ANOVA) followed by the Bonferroni test was used to compare means, and the Kruskal–Wallis and Dunn tests were used to compare medians. *P* < 0.05 was considered significant^43^.

## Supplementary information

Supplementary information
