# Supplementary material for: 5-Fluorouracil Induces Enteric Neuron Death and Glial Activation During Intestinal Mucositis via a S100B-RAGE-NFκB-Dependent Pathway
Source: Sci Rep. 2019 Jan 24;9:665. doi: 10.1038/s41598-018-36878-z (PMC6345953; doi:10.1038/s41598-018-36878-z)
Supplement: Supplementary file 1 — Supplementary information [file 41598_2018_36878_MOESM1_ESM.pdf]

Supplementary information

5-Fluorouracil Induces Enteric Neuron Death and Glial Activation During Intestinal Mucositis via a S100B-RAGE-NFκB-Dependent Pathway

Deiziane V.S. Costa[1,2], Ana C.O. Bon-Frauches[3], Angeline M.H.P. Silva[1], Roberto C.P. Lima-Júnior[2], Conceição S. Martins[1], Renata F. C. Leitão[1], Gutierrez B. Freitas[1], Patrícia Castelucci[4], David T. Bolick[5], Richard L. Guerrant[5], Cirle A. Warren[5], Vivaldo Moura-Neto[3], and Gerly A.C. Brito[1,2*]

[1]Department of Morphology, Faculty of Medicine, Federal University of Ceará, Fortaleza, Ceará, Brazil.

[2]Department of Physiology and Pharmacology, Faculty of Medicine, Federal University of Ceará, Fortaleza, Ceará, Brazil.

[3]Paulo Niemeyer Brain Institute, Federal University of Rio de Janeiro, UFRJ, Rio de Janeiro, Brazil.

[4]Department of Anatomy, University of São Paulo, São Paulo, SP, Brazil.

[5]Department of Infectious Diseases, University of Virginia, Charlottesville, VA, USA.

*Correspondence and requests for materials should be addressed to Gerly A. C. Brito (E-mail: gerlybrito@hotmail.com).

Supplementary Figures

Figure S1

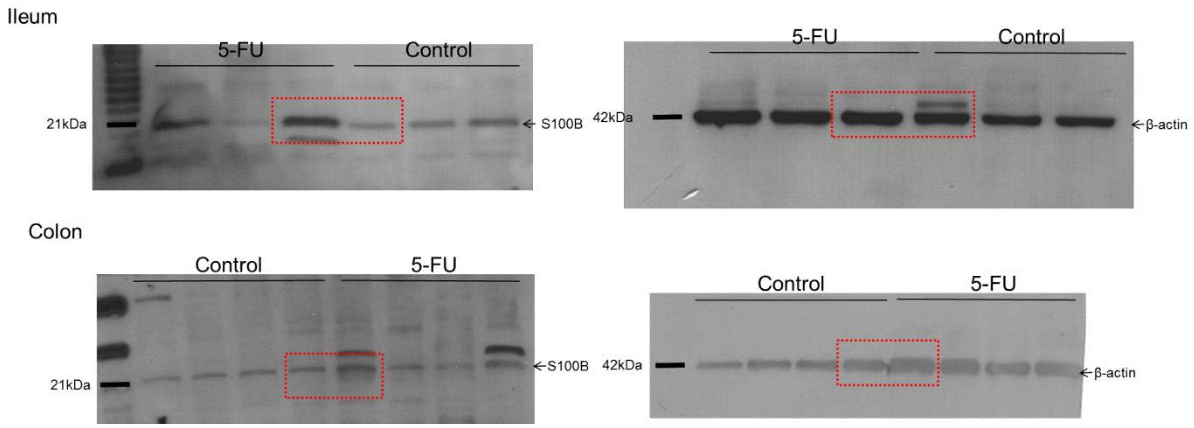

5-FU increases S100B protein expression in the ileum and colon of mice. Uncropped image of the blot used in Fig. 1A. Segments of ileum and colon from mice receiving only saline (Control) or 5-FU were homogenized in RIPA lysis buffer followed by western blot detection using an anti-S100B (Santa Cruz Biotechnology, 1:100), anti-β-actin (Millipore, 1:500), a loading control, and anti-goat (Invitrogen; 1:1000) and anti-rabbit (Invitrogen; 1:1000) secondary antibody. The dotted rectangle represents the cropped image used in Fig. 1A.

Figure S2

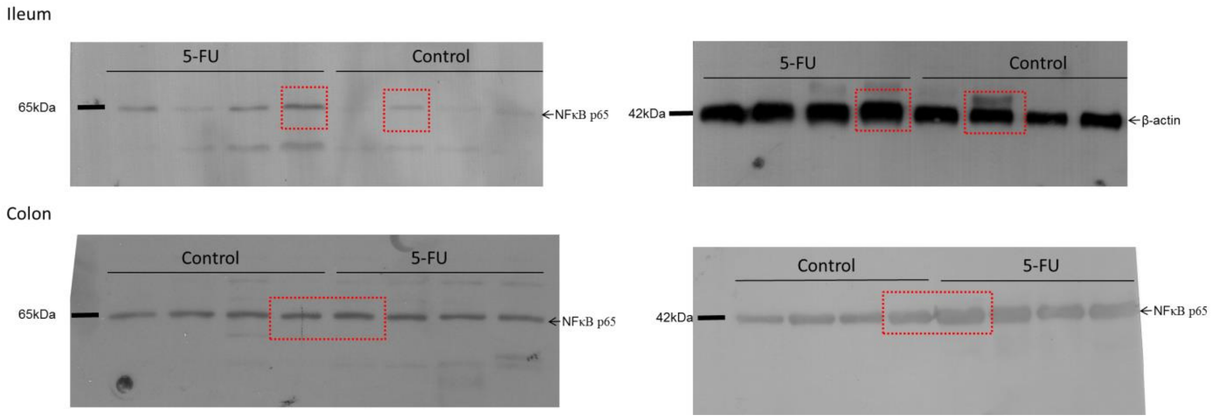

5-FU increases S100B protein expression in the ileum and colon of mice. Uncropped image of the blot used in Fig. 1B. Segments of ileum and colon from mice receiving only saline (Control) or 5-FU were homogenized in RIPA lysis buffer followed by western blot detection using an anti-NFκB p65 (Santa Cruz Biotechnology, 1:200), anti-β-actin (Millipore, 1:500), a loading control, and anti-rabbit (Invitrogen; 1:1000) secondary antibody. The dotted rectangle represents the cropped image used in Fig. 1B.

Figure S3

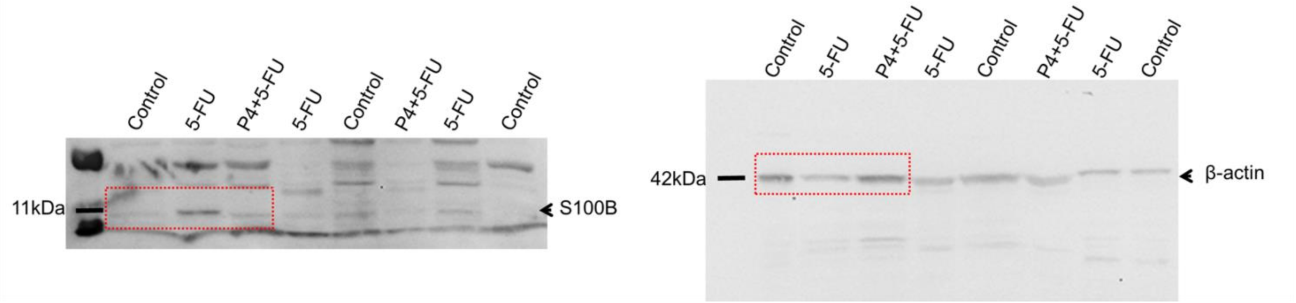

S100B inhibitor decreases S100B protein expression in the jejunum. Uncropped image of the blot used in Fig. 3F. Jejunum segments from mice receiving only saline (Control), 5-FU or 5-FU and S100B inhibitor (P4+5-FU) were homogenized in RIPA lysis buffer followed by western blot detection using an anti-S100B (Santa Cruz Biotechnology, 1:100), anti-β-actin (Millipore, 1:500), a loading control, and anti-goat (Invitrogen; 1:1000) and anti-rabbit (Invitrogen; 1:2000) secondary antibody. The dotted rectangle represents the cropped image used in Fig. 3F.

Figure S4

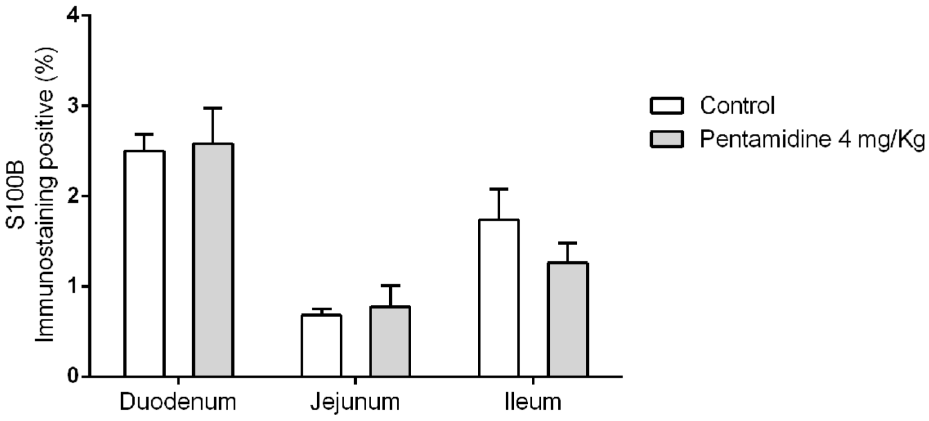

Effects of Pentamidine on S100B expression in the small intestine. Graphs represent the mean ± SEM of the percentage of S100B immunopositive area in the small intestine (duodenum, jejunum and ileum) related to total tissue in 5 microscope fields per mouse from 4 mice in each group, quantified using Photoshop. #*P*< 0.01 versus control group, Student's t test.

Figure S5

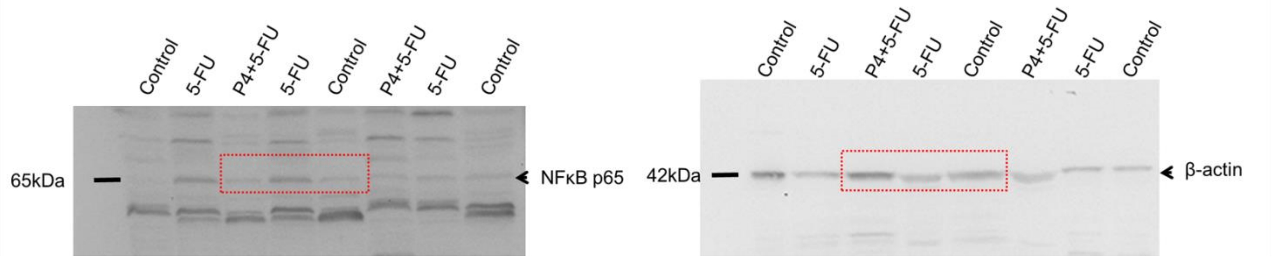

S100B inhibitor decreases NFκB p65 protein expression in the jejunum. Uncropped image of the blot used in Fig. 5D. Jejunum segments from mice receiving only saline (Control), 5-FU or 5-FU and S100B inhibitor (P4+5-FU) were homogenized in RIPA lysis buffer followed by western blot detection using an anti-NFκB p65 (Santa Cruz Biotechnology, 1:200), anti-β-actin (Millipore, 1:500), a loading control, and anti-rabbit secondary antibody (Invitrogen; 1:1000). The dotted rectangle represents the cropped image used in Fig. 5D.

Figure S6

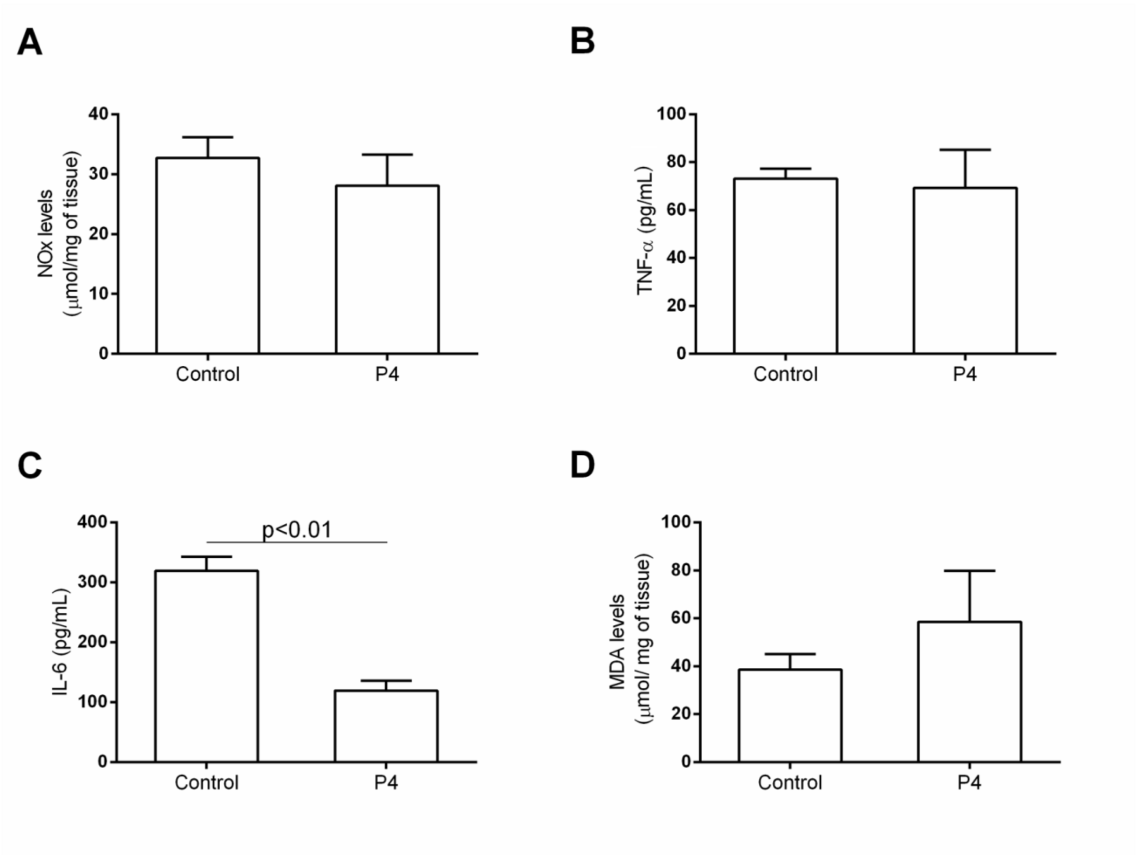

Effects of Pentamidine on pro-inflammatory mediators levels and oxidative stress. (A) Nitrite and nitrate levels were evaluated by the Griess method. (B) TNF-α and (C) IL-6 levels were measured by ELISA. (D) MDA levels were evaluated by the TBARS method. Bars represent mean ± SEM of 6 mice in each group. *P*< 0.01 versus control group, Student's t test.
